# Place Attachment and Views on Tree Management

**DOI:** 10.3389/fpsyg.2021.639830

**Published:** 2021-06-03

**Authors:** Daria Paniotova-Maczka, Piotr Matczak, Piotr Jabkowski

**Affiliations:** ^1^Faculty of Psychology and Cognitive Science, Adam Mickiewicz University, Poznań, Poland; ^2^Faculty of Sociology, Adam Mickiewicz University, Poznań, Poland

**Keywords:** place attachment, ecosystem services, rural vs. urban, the decision of cutting off trees, landowner vs. municipality, relational values, trees management

## Abstract

Few studies have investigated relational environmental views of different stakeholder groups. In this study, we investigated how residents of rural and urban municipalities view the management of trees (who should decide about trees’ removal – the landowner, or the municipality), which provides a various range of ecosystem services and the extent that place attachment as a relational variable affects these views. The analysis was based on 231 questionnaires conducted in two Polish municipalities: one rural (Nysa) and one urban (Racibórz). Data were analyzed using statistical methods including logistic regression models for analyzing factors impacting the main research question. Our investigation showed that both place attachment involving public good sentiments and the perception of ecosystem services provided by trees, that are related to private interests significantly impacted views on tree management. In rural areas the opinion, that the municipality should decide to remove trees was positively associated with a place attachment. For residents of urban areas (Racibórz), the strength of place attachment was not related to the perception of tree removal, but it was related to the perception of trees’ cultural benefits. We argue that considering psychological variables related to the tree management issues could help avoid conflicts.

## Introduction

Trees are an essential part of the environment. They provide several ecosystem services (ES), which include the direct benefits that natural environment delivers to people and positively affects human well-being (Millennium Ecosystem Assessment, 2005; Haines-Young and Potschin, 2013). Three main types of ES are distinguished: (1) provisioning (e.g., a supply of fruits and nuts, wood, and leaves); (2) regulation and maintenance (e.g., a source of oxygen, protection against the wind, and a positive effect on health as by producing phytoncides – antibacterial substances released into the air by leaves, flowers or bark); and (3) cultural (e.g., space aesthetics, place of recreation, and strengthening interpersonal relationships). As such, ES secure livelihoods and quality of life for individuals and communities. Despite this, tree cover loss is observed, caused by development pressure especially in urban areas (Nowak and Greenfield, 2018, 2020). In rural areas, trees decline occurs due to the changes in agriculture practices and difficulties with trees management, planting, cultivation, and cutting off (Suchocka et al., 2019).

Since trees provide combinations of ES, for particular private individual and publicly – for groups, their management is complex and can be organized and distinguished using two main tree management models. Firstly, public administration (municipal, regional, or national) has the decisive role and supports trees, forests and green areas as a public good, where all members of the community can benefit. Alternatively, individual owners assume that residents care for their own trees, gardens, orchards, and forests. They control and gain profits from their trees. They also contribute to public goods provision as their trees provide, for instance, scenic beauty that is valued by the community. Trees share characteristics of public goods. The theory of public goods suggests that leaving the management of public goods to individuals leads to under-provision (Mincey and Vogt, 2014). Common-pool resources are exhaustible and not prohibited from use (Schlager and Ostrom, 1992), which leads to their overexploitation. Therefore, regulations are imposed on human activities to define the property rights and rules of trees management in order to protect the environment. Public authorities (e.g., the municipality offices) need to take care of the green areas in order to secure the sustained provision of the public goods stemming from trees. In many countries, regulations concerning tree management on private land are in place, restricting tree removal by owners. Considering the complexity of ES provided by trees, these regulations need to count on residents’ and owners’ tree management views and motivations. Restrictions imposed on owners involve substantial trade-offs between the rights of a private owner and the need to sustain the quality of public goods. In Poland, the country of this study, forests’ management is regulated by a specific law, defining the rights and obligations of the owners. This study concerns trees which are not part of the forests. The non-forest tree are also under the provision of the national regulation that specifies that a permit need to be obtained for removing trees in public (e.g., municipal) and private land. It does not apply to fruit and small trees but in most cases the permit to remove a tree is required. For most cases the permits are issued by the municipalities’ administrations and involve compensation. This law was suspended for some month in 2017 igniting a discussion about tree protection and management.

A balance between residents’ attitudes toward the ES and the regulations securing trees as public goods is an essential issue from both scientific and practical perspectives. Tree policies should account for peoples’ tree-related values. The value of nature is conceptualized in several ways (Cundill et al., 2017). Nature is viewed dichotomously: via instrumental values (i.e., what we can “do” with what we have) and via the intrinsic values (i.e., what we consider essential). However, people rarely make choices using this simple distinction, and often consider both their relationship with nature and with others (Chan et al., 2016). This relationship is mediated through a variety of factors, including social norms, social cohesion, cultural identity, and policies (Díaz et al., 2015; Chan et al., 2016). These factors refer to the concept of relational values (Chan et al., 2018), which are “the values that are imbedded in desirable (sought after) relationships, including those among people and between people and nature” (Díaz et al., 2015; Stålhammar and Thorén, 2019). Relational values are not present in naturally occurring objects such as trees, but are the derivative of relationships to them. One of these relational values is place attachment (Cundill et al., 2017). People can look at nature differently depending on their emotional connection with nature. If nature is present directly next to their place of residence, then the emotional connection with the place of residence will include natural objects that are located directly on the territory of the residence. Place attachment can be an important factor that mediates individual views on trees management. For example, it motivates individuals to spend more time outdoors and to protect the landscape that is directly related to ES provided by trees. Relational values shape individuals’ relationship with nature.

Although, relational values in the context of nature protection and sustainability are a topic of growing importance (Chan et al., 2018), these contributions are mostly conceptual (Knippenberg et al., 2018; Muradian and Pascual, 2018). De Vos et al. (2018) call for studies on the relational values of multiple, disaggregated stakeholder groups in their assessment, as it would help to highlight power imbalances and trade-offs in prioritizing relational values of various groups and subgroups.

In this paper, we address this need. Specifically, we look how place attachment can support an understanding of how two different groups of residents (rural and urban) differ in their view of the tree management. We investigated whether stronger place attachment is correlated with a preference for private- or public-led tree management. We also test whether views on tree management can be influenced by the demographic and environmental variables, such as the perceived benefit of trees. This study is the first investigation to address place attachment’s influence on rural and urban views on tree management.

## Literature Review

### Perception of the Benefits of Trees by Residents in Rural and Urban Areas and Involvement in Tree Management

Recent research shows an unclear picture of resident views on tree benefits and tree management. In general, people who see the benefit of trees are more likely to feel responsible for managing trees, while people who think that trees create problems are not willing to participate in urban tree management (Moskell and Allred, 2013; Breger et al., 2019). Moreover, the perception of the benefits of trees depends on ES they provide (i.e., the attributes of trees). For example, trees that tend to fall or drop many leaves are perceived as less useful overall (Camacho-Cervantes et al., 2014; Fernandes et al., 2019). Nevertheless, people more often note the benefits of trees, rather than their drawbacks, and tend to prefer more trees in cities. Tree-related benefits are also more noticeable if a tree is closer to a house as the tree is perceived as a means of directly improving air quality. Abd Kadir and Othman (2012) identify three areas on which one can take advantage of trees: (1) the physical sphere (e.g., the reduction of the greenhouse effects, the purification of air from small polluting particles, the provision of shade, the surfaces shielded from electric breaks, the management of stormwater, the increase in property value, and the aesthetic beauty); (2) the social sphere (e.g., the benefits of trees are related to the security that trees can offer such as slower traffic on streets where trees are planted); (3) the economic sphere (e.g., trees on land increase the value of a house, due to benefits including shade, contributing to energy-savings, and reducing electricity bills. Few studies concern residents’ tree management views in rural areas (Dei, 1992; Vermeulen, 1996). According to Chambers and Leach (1989), trees offer savings and security for the rural poor. Trees in rural areas, especially poor ones, play an essential role in providing resources and contributing to economic security. Trees can be a crucial economic resource. In India, trees are used to provide collateral when applying for a loan, so that if a person did not repay their loan, the bank could remove their trees valued equivalent to the loan amount and interest. Trees could also pay off loans and repay debts (Chambers and Leach, 1989). Therefore, it was the right to dispose of trees in the remote territory, that increased their creditworthiness, and protected natural disasters such as flooding. Studies have shown that people living in rural areas perceive trees as offering many positive and negative contributions (Blanco et al., 2020). The benefit of firewood was especially emphasized as a means to save energy costs. The importance of intangible ES, such as scenic value and originality of landscape as well as traditional land-use practices, was also noted. For example, villagers harvest firewood and collect fruits, nuts and mushrooms not only to meet material needs, but also to maintain certain traditional practices and social interactions. Of the negative ES, there is a physical obstacle to mechanization, the difficulty of matching mechanized agriculture with trees, which is associated with cutting down trees, and additional expenses associated with damage caused by trees.

These studies show that perceived tree benefits vary depending on the area. Nonetheless, the way in which these differences affect the views on tree management, including who decides on cutting trees, has not been investigated. Moreover, previous studies show that protecting natural objects such as trees or changing them (including cutting down) can lead to conflicts between the authorities and private landowners (Götmark, 2009; Devine-Wright and Howes, 2010).

### Place Attachment and Relational View on Nature

Place attachment can be considered in three conceptual dimensions: (1) the personal dimension, where attachment to a place is considered in the context of the place’s meaning; (2) the psychological dimension, where attachment includes an emotional, cognitive and/or behavioral component; and (3) the place dimension, where attachment to a given place is considered in the context of components and characteristics (e.g., social and physical) of that place (Brown and Raymond, 2007; Scannell and Gifford, 2010). Along with the concept of place attachment are the concepts of the significance of a place (Stedman, 2003; Jorgensen and Stedman, 2006), one’s identification with a place (Hernández et al., 2007; Lewicka, 2008), and the dependence on a place (Trentelman, 2009). Some studies also consider three concepts as predictors of the sense of a place (Jorgensen and Stedman, 2006). Place attachment is also viewed as an integral part of identification with a place (Low and Altman, 1992; Twigger-Ross and Uzzell, 1996; Mazumdar et al., 2000; Stedman, 2002). In this study, we focused on an emotional connection with the place for three primary reasons. Firstly, it is a component of the psychological dimension of place attachment. Secondly, the emotional component is the strongest indicator of attachment. Finally, the emotional component of place attachment is often a component in other approaches that explains a person’s relationship with a place, such as identification with a place and the meaning of a place (Perkins and Long, 2002; Lewicka, 2008).

We assume that attachment to a place can indirectly influence tree management opinions. Attachment to place is also a manifestation of a relational view of nature and contributes to the development of relational connections with nature. This connection appears primarily as the interest in nature. It is a responsibility for nature and care for it. Since attachment to a place can be of different strengths, these relationships will consequently display various strengths. Some social and demographic factors, such as house ownership, length of stay in one locality, age, gender, education, and income (Anton and Lawrence, 2014) can be predictors of the place attachment strength. A sense of local community and local social contacts (Buchecker and Frick, 2020) are also predictors of stronger place attachment.

Research suggests that, in general, people living in rural areas are more firmly attached to their places of residence than are people living in urban areas (Lewicka, 2011; Anton and Lawrence, 2014; Verbrugge and van den Born, 2018). Gosling and Williams (2010) argue that a connection with nature plays a significant role in preserving green spaces in rural areas among farmers. Those farmers who felt a greater connection with nature were inclined to preserve trees on their territory. There are several predictors of place attachment strength, such as owning one’s own home and their type of settlement. These factors are correlated with views on tree management.

### Place Attachment and Involvement in Activities Related to the Place

Previous studies show that place attachment can play a key role in shaping attitudes toward planning a change of place, an interest in managing a place, and involvement in its affairs (Davenport and Anderson, 2005; Walker and Ryan, 2008; Buta et al., 2014; Van Veelen and Haggett, 2016). However, there is a lack of knowledge surrounding the connection between place attachment’s strength and preferences concerning the environment (e.g., concerning views on tree management). On the one hand, studies (Mohapatra and Mohamed, 2013) show that, the people who have a stronger place attachment more often support of pro-environmental measures in relation to natural objects (e.g., parks). On the other hand, a stronger place attachment can contribute to supporting the status quo of a place and is a cause of resistance to changes associated with a place (Devine-Wright and Howes, 2010; Bonaiuto et al., 2016). This divergence may be due to two main factors: services provided by the place and the rural or urban location. The difference in the perception of the services provided by the place influences the strength of place attachment and can play a key role in shaping preferences around tree removal decisions.

Studies on place attachment and involvement in the management of these places carried out in urban areas, concerning parks, squares, and city forests, show that people who are more attached to natural places willingly support nature conservation when a natural place is their place of recreation, psychological restoration, or a setting for sports (Mohapatra and Mohamed, 2013). In rural areas, landscapes, including trees, are often perceived as an economic resource that provides residents with certain benefits. Previous studies in rural areas show that a high level of place attachment and a high level of positive attitude toward landscapes was a significant obstacle to planned changes to landscapes. When individuals are comfortable with a place, they do not support change to that place (Park and Selman, 2011; Chappell et al., 2020). Studies by Walker and Ryan (2008) show a strong positive correlation between attachment to the rural landscape and support for activities aimed at protecting agricultural soils. In urban environments, residents tend to support landscape changes if they are of benefit (Von Wirth et al., 2016). In general, changes are more opposed in the countryside than in urban areas. In rural areas, people are reluctant to support change that threatens their identity or limits their economic opportunities. Cutting down trees means a change in place and landscape. Therefore, place attachment will influence the perception of place change, and in the present case, place attachment impacts the perception of tree removal.

Previous studies also show that different place attachment includes support for specific changes. Inhabitants of rural areas who have recently moved to an area are reluctant to support changes in the social and economic sphere (e.g., community self-support, availability of shops), however, they support changes in the natural environment (place-protective behavior). Conversely, inhabitants who live longer in an area do not support changes in the natural environment, and instead support social and economic changes (Zwiers et al., 2018).

### The Conceptual Model of the Study

**FIGURE 1:**
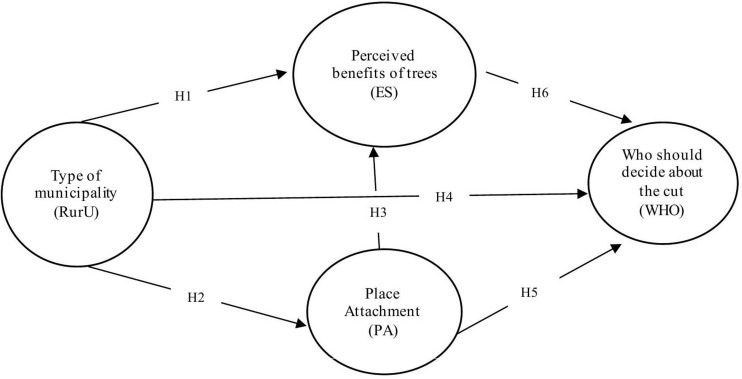
The conceptual model of the study.

In this study, we verify the impact of perceived ES provided by trees on views regarding preferred type of tree management, specifically who should decide about tree removal on a private land: the owner or the municipality. We treat place attachment as a moderating variable. Moreover, rural vs. urban municipality type was considered a factor influencing both place attachment and the perceived benefits of trees. Finally, possession of trees is considered a variable that may influence the perception of benefits and the preferred type of tree management. The research model is shown in Figure 1.

Place attachment (PA) is a latent construct understood as a respondent’s bond to the place of residence. The perceived benefit of trees (ES) is understood as a declared respondent’s tree-derived utility. Moreover, rural and urban areas (RurU) denote respondents’ place of living in the village or the city. Finally, the variable who (i.e., whether the owner or the municipality should decide about tree removal; WHO) denotes the expected decision-maker.

We have established the following hypotheses:

H1. People living in urban/rural areas differ in the perceived benefits of trees. Specifically, people living in rural areas prefer provisioning benefits of trees, while people living in urban areas prefer those that are cultural.
H2. People living in rural areas are characterized by stronger place attachment compared to people living in urban areas.
H3. People who are more attached to a place perceive more benefit from trees than people who are less attached.
H4. People living in rural areas support the opinion that the owner should decide to remove trees from the land.
H5. In rural areas, support for the opinion that the municipality should decide about tree removal is positively associated with the place attachment and negatively associated with provisioning benefits of trees.
H6. In urban areas, supporting the opinion that the municipality should decide about tree removal is positively correlated with the place attachment and the perception of cultural benefits of trees.

## Materials and Methods

### Sampling Characteristics

We surveyed respondents in two Polish municipalities, Racibórz and Nysa, in June 2019. The municipalities were selected purposively, as Racibórz is urban, and in Nysa we collected data only in the rural areas. Racibórz (55 thousand inhabitants) and Nysa (58 thousand inhabitants) represents medium size Polish municipalities. They were selected as “typical” in terms of greenspace coverage.

Data were collected via computer-based website questionnaires (CAWI). Respondents were asked to indicate trees that are relevant to them on the map of their municipalities, and to attribute ES to the trees they had indicated. Indicated trees could be both in private and in public land and ES were attributed to each tree separately. One or more ES could be attributed to a tree. Respondents were then asked to answer the question of who should decide about tree removal in private land. The information on the survey was advertised in mass media and the web pages of both municipalities. However, due to an insufficient number of completed questionnaires in Nysa, we also carried out face-to-face interviews. In these cases, the respondents filled in the questionnaire on laptops provided by a researcher (CAPI). After eliminating questionnaires with missing responses, a total of 231 questionnaires were included in the analysis (135 from Racibórz, 96 from Nysa). Table 1 provides information about the socio-demographic profile of respondents.

**TABLE 1:** Sample distribution of respondents’ socio-demographics.

**Demographic**	**Frequency**	**Percent**
Gender		
Male	96	40.7
Female	140	59.3
Age		
Up to 20	37	15.7
21–30	52	22.0
31–40	55	23.3
41–50	32	13.6
51 and older	60	25.4
Level of education		
Primary	19	8.1
Secondary	38	16.1
Post-secondary	79	33.5
Higher	98	41.5

### Dependent Variable

Our dependent variable includes answers to the question: “Who should decide about tree removal on private land?,” with three options for respondents to select: (i) the landowner; (ii) the municipality office or other offices, depending on the purpose of removal; and (iii) the landowner, except in specific situations – questionnaire is attached together with the data set<.

### Independent Variables: Covariates and Factors

The following independent variables were included in the analysis: (1) the type of municipality (a binary variable: rural/urban); (2) place attachment (measured by a latent variable that determines the strength of an emotional connection with the place of residence, α = 0.869). For the purposes of the study, we took the scale by Lewicka (2011), which consists of nine questions about people’s feelings toward their place of residence. Participants replied to how much they agree with a given statement (e.g., “I miss the place when I am not here”). These response options ranged from one to five and indicate the strength of respondents’ feelings about the place of residence – questionnaire is attached together with the data set; and (3) perceived benefits of trees, where respondents chose one or more of 18 ecosystem services indicating benefits brought by trees that they pointed out on the map (a binary variable: yes/no). Three types of ES were also considered in the analysis: (1) provisioning (fruits and nuts, economic benefits, timbers, branches and leaves); (2) regulation and maintenance (wind protection, noise control, positive effects on health and wellness, animal habitat and food source, air and soil humidification, air purification, snowdrifts, sun protection); and (3) cultural (tree as a witness to cultural history, contribution to the aesthetics of space, educational usefulness, a sense of intimacy provision, separating from neighbors, strengthening interpersonal bonds, and recreation space). We relied on earlier studies and compiled a preliminary list of ES relevant to trees. It was included to the questionnaire, which was further tested. Eventually, 18 ES were mentioned and the option “other” was also available. Indicated trees could be both on private and on public land and ES were attributed to each tree separately. Furthermore, we included in the questionnaire additional variables such as possession of trees and demographic characteristics of respondents (e.g., gender, age, and level of education).

### Statistical Procedures for Data Analysis

All statistical procedures implemented in this study have been documented in the replication syntax file provided in the online supplementary materials; hence we do not describe here all the procedures implemented in the paper, especially those well-known like *t*-test, Pearson correlations or linear regression analysis. We only specify logistic regression models for analyzing factors impacting on the view on who should make decisions about tree management.

We start with dichotomizing the dependent variable by merging two separate categories of respondents who indicated that “only owner” or “owner except in specific situations” should decide to remove trees. This decision was made due to very few cases in which respondents selected the latter category. Next, we specified a Logistic Regression model to explain the relationship between the dependent variable (who should decide about tree removal) and a subset of the independent variables (place attachment (PA), perceived benefits of trees: provisioning (ES1), regulation and maintenance (ES2), cultural (ES3), municipality type (rural/urban; RurU). However, we found collinearity between the two independent variables “regulation and maintenance” and “cultural”. Therefore, we finally conducted the regression analysis using only two variables concerning ES1 and ES3. We also controlled for gender and age.

A detail specification of the regression model is as follows. Let us denote by *TREE*_*i*_ an outcome dichotomous variable, where *E*(*TREE*_*i*_ = 1) = π_*i*_ is the probability of indicating by respondent *i* that municipalities should decide on cutting down trees. We used a logit link function (based on the natural logarithm), where (1) the logit coefficient ${}_{i}=\text{log}\,\left(\frac{\pi_{i}}{1-\pi_{i}}\right)$ is the log of the odds of the event *TREE*_*i*_=1 as opposed to *TREE*_*i*_=0. We ran two logistic regression models: Model 1 with the vector of regression coefficients to assess the impact of particular covariates on the probability of indicating that municipality should decide on cutting down trees; and Model 2 with interactions to assess whether the impact of the rural/urban place of living on tree management views is mediated by other covariates. The specification of Model 1 is as follow:

$$\begin{array}{c} n_{i}=\beta_{0}+\beta_{1}\,R\,u\,r\,U_{i}+\beta_{2}\,g\,e\,n\,d\,e\,r_{i}+\beta_{3}\,a\,g\,e_{i}+\beta_{4}\,P\,A_{i}+\\ \beta_{5}\,E\,S\,1_{i}+\beta_{6}\,E\,S\,3_{i}. \end{array}$$

The regression equation for Model 2 is in turn as follows:

$$\begin{array}{c} n_{i}=\beta_{0}+\beta_{1}\,R\,u\,r\,U_{i}+\beta_{2}\,g\,e\,n\,d\,e\,r_{i}+\beta_{3}\,a\,g\,e_{i}+\beta_{4}\,P\,A_{i}+\beta_{5}\,E\,S\,1_{i}+\\ \beta_{6}\,E\,S\,3_{i}+\beta_{7}\,R\,u\,r\,U_{i}*P\,A_{i}+\beta_{8}\,R\,u\,r\,U_{i}*E\,S\,1_{i}+\\ \beta_{9}\,R\,u\,r\,U_{i}*E\,S\,3_{i} \end{array}$$

where:

∘ β is a vector of regression coefficients.

In general, we looked for factors impacting the view on who should make decisions about tree management, and we also consider whether the impact of the type of municipality is mediated by other variables.

## Results

The perceived benefits of trees’ provisioning services are valued less by the residents of Racibórz (*M* = 0.094, SD = 0.21) than by the residents of Nysa (*M* = 0.28, SD = 0.26). The difference is statistically significant when univariate *t*-test was used to analyse the difference (*t* = 5.65, *p* < 0.001), and remained significant after controlling for gender and age (*t* = 5.49, *p* < 0.001). The difference between the residents of Racibórz (*M* = 0.33, SD = 0.24) and Nysa (*M* = 0.32, SD = 0.28), in terms of the perception of cultural benefits from trees did not reach significance (*t* = 0.22, *p* > 0.05). Therefore, concerning the hypothesis 1 (H1) – “People living in urban and rural areas will differ in perceiving the benefits of trees. People living in rural areas prefer (1) provisioning benefits of trees, while people living in urban areas prefer those that are (2) cultural ones,” our analysis shows that the difference in perception of ES by the residents of two municipalities is partially confirmed. The average value of the strength of place attachment is lower among the inhabitants of Racibórz (*M* = 0.65, SD = 0.79) than among the inhabitants of Nysa (*M* = 1.22, SD = 0.84). The analysis shows that the difference is statistically significant both for univariate analysis (*t* = 5.22, *p* < 0.001) and after controlling for gender and age (*t* = 4.15, *p* < 0.001). Concerning the hypothesis 2 (H2) – “People living in rural areas are characterized by stronger place attachment comparing to people living in urban areas” our analysis shows that the difference in the strength of place attachment of the residents of two municipalities fully confirmed.

A Pearson’s correlation and partial correlation controlling for gender and age was performed to examine the relationship between the level of place attachment and the level of perceived tree benefits. The results show that the relationship between the level of place attachment and the level of perceived tree benefits is statistically significant for the provisioning benefits of tress (correlation coefficient: *r* = 0.19, *p* < 0.05; partial correlation coefficient: *r* = 0.18, *p* < 0.05). The relationship between the strength of place attachment and the perception of cultural benefits of trees was not statistically significant (*r* = 0.07, *p* > 0.05). Thus, concerning the hypothesis 3 (H3) – “People who are more attached to a place (strength of place attachment) will see more benefits from trees than people who are less attached” our analysis shows that the relationship between strength of place attachment and perceived benefits from trees is partially confirmed.

The analysis also confirmed that the residents of Nysa more often support the opinion that the decision to remove trees should be made by the owner of the territory (78.1%) compared to the residents of Racibórz (56.2%). The difference is statistically significant (Fisher’s Exact Test = 0.001). Therefore, concerning the hypothesis 4 (H4) – “People who live in the rural areas more often than those living in urban areas prefer that the owner of the territory on which the tree grows should decide on the cutting of the tree,” our analysis shows that the difference in preferences about who should decide on the cutting of the tree of residents in two municipalities is confirmed.

However, the question arises whether the difference between rural and urban municipalities are still significant in this respect when we control for gender, age, place attachment, cultural- and provisioning benefits of trees. Thus we applied a logistic regression models in accordance with the specifications outlined earlier in the paper to determine factors influencing the opinion of Racibórz and Nysa inhabitants about who should decide to remove trees; the municipality or the owner of the land.

Concerning the fifth and the sixth hypotheses, (H5) “In rural areas, support for the opinion that the municipality should decide about tree removal is positively associated with the place attachment and negatively associated with provisioning benefits of trees,” (H6) “In urban areas supporting the opinion that the municipality should decide about tree removal is positively related with the place attachment and the perception of cultural benefits of trees” our analysis shows the mixed results.

Firstly, the results of both regression models demonstrate that, in general, the opinion that the municipality should decide to remove trees is positively associated with the perception of the cultural benefits of trees (Model 1: *B* = 1.34, *p* < 0.05; Model 2: *B* = 2.17, *p* < 0.05) and negatively associated with the perception of the provisioning benefits of trees (Model 1: *B* = −2.4, *p* < 0.05; Model 2: *B* = −1.47, *p* > 0.05) – Table 2. However, there are no differences between rural and urban municipalities in this respect which is demonstrated by a non-significant effect of interactions between the type of municipality and cultural and provisioning benefits of trees. On the other hand, the positive effect of interaction between type of municipality (Rural = 1) and place attachment in Model 2 (*B* = 0.97, *p* < 0.1) supports the hypothesis that in rural areas opinion that the municipality should decide about tree removal is positively associated with the place attachment. Note that gender and age of respondents have no significant impact on the opinion on who should decide to cut down trees.

**TABLE 2:** Logistic regression results.

**Covariates**	**Model 1**			**Model 2**		
	**B**	**SE.**	**Exp(B)**	**B**	**SE.**	**Exp(B)**
RurU: Type of municipality (Rural = 1)	−0.562	0.384	0.570	−0.775	0.845	0.461
Gender of the respondent (Male = 1)	0.461	0.324	1.602	0.384	0.333	1.468
Age	−0.018	0.012	0.982	−0.018	0.012	0.982
PA: Strength of the place attachment	0.225	0.200	1.251	−0.081	0.247	0.923
ES1: Perceived benefits of trees: provisioning	−2.384*	0.837	0.092	−1.466	1.108	0.231
ES3: Perceived benefits of trees: cultural	1.337*	0.654	3.806	2.166*	0.884	8.724
RurU * PA				0.965^	0.531	2.625
RurU * ES1				−2.067	1.737	0.127
RurU * ES3				−1.867	1.414	0.155
Constant	−0.798	0.730	0.274	−0.778	0.787	0.459
**Fit statistics**						
-2 Log-likelihood	254.387			246.766		
Nagelkerke R Square	0.158			0.200		
Hosmer and Lemeshow Test	χ^2^ 6.4; df = 8; *p* = 0.602			χ^2^ 5.7; df = 8; *p* = 0.678		

^*p*-value < 0.1; *p-value < 0.05.

## Discussion

Our study brings insights into a relational view of nature, manifested via place attachment. We tested whether place attachment could be a significant indicator of the opinion about tree management, and specifically on the issue of who should decide to remove trees residents or – the municipality.

Our results support the results of previous findings, that the rural/urban distinction has a significant impact on perceptions surrounding tree management (Jones et al., 2013; Blanco et al., 2020). Residents of the rural area (the municipality of Nysa), more often support the opinion that the owner of the land should decide about tree removal compared to residents of the urban area (the municipality of Racibórz).

The impact of the rural/urban place of living on tree management views is mediated by place attachment. In the rural area (Nysa), a stronger emotional connection with the place of residence implies residents’ support municipalities deciding to remove trees. This may be due to the fact that a stronger place attachment in a rural area creates a greater sense of security and, as a consequence, a stronger trust in local authorities (Verbrugge and van den Born, 2018; Song et al., 2019; Peng et al., 2020), however, as we did not explicitly test this reasoning through our study, this offers an opportunity for future research. However, stronger emotional connections to the place of residence within urban area (Racibórz) does not lead to more support for the municipality’s decisions to remove trees.

Our study results regarding the difference in place attachment’s strength between rural and urban inhabitants are consistent with previous studies, indicating a tendency of greater emotional place attachment of rural inhabitants (Anton and Lawrence, 2014). Apart from this, our study results show that people who have greater strength attachment to their place of residence perceive more benefits from trees in terms of provisioning benefits of trees, regardless of the type of locality (rural vs. urban). In general, this is somewhat consistent with previous research that shows that a generally greater sense of place, identification with a place, and attachment to objects of nature increases involvement in activities about that place and increases interest in that place (Ryfield et al., 2019; Faccioli et al., 2020). Therefore, it can be assumed that people who have a higher place attachment see more benefit from the place and will be more likely to get involved in the place’s affairs. More substantial involvement in these affairs can be associated with more interest in changes of place. However, how such a tendency would apply to the formation of an opinion about who should decide about tree removal remains to be investigated in further research. Besides, investigate the relationship between the strength of place attachment (identification with a place, attachment to the landscape) and openness to landscape changes in the case of trees removal (Walker and Ryan, 2008; Von Wirth et al., 2016; Verbrugge and van den Born, 2018). Future research may also check whether place attachment is related to the sense of trust of the administration that operates in a given place and how it is applied to the formation of an opinion about who should decide on tree removal.

Another mediating variable was the perception of ES provided by trees. Our research shows that the inhabitants of urban and rural areas differ in terms of the perception of the benefits provided by trees. The rural area inhabitants (Nysa) perceive the benefits from trees more in terms of provisioning than the inhabitants of the urban area (Racibórz). This is inconsistent with the previous results, where urban inhabitants valued provisioning ES and rural residents valued regulating ES (Yang et al., 2019). The same study by Yang et al. revealed the lack of differences between urban and rural residents concerning the perception of cultural benefits of trees. The differences between our study and the Yang et al.’s may result from the cultural, landscape, climatic and spatial differences between rural and urban areas in Poland and China. However, checking this would require further and more extensive research.

In general, the opinion that the municipality should decide to remove trees is positively associated with the perception of trees’ cultural benefits and negatively associated with the perception of the benefits of providing resources from trees. However, in this respect, there is no difference between rural and urban municipalities.

Both place attachment involving public good sentiments and the perception of ES provided by trees that are related to private interests significantly impacted views on tree management. The factors reflect two overall trends. In rural areas (Nysa), the opinion that the municipality should decide to remove trees is positively associated with a place of attachment. For residents of urban areas (Racibórz), the strength of place attachment was not related to the perception of tree removal, but it was related to the perception of trees’ cultural benefits.

Living in rural or urban areas reflects a difference in views on tree management. There is a higher degree of place attachment in rural areas, more perceived ES provisioning, and a stronger view that landowners should decide about tree removal. Those in rural areas that perceive the provisioning benefits of trees tend to support the view that the landowner should decide to cut down trees. Trees that are useful in bearing fruit and providing wood strengthen opinions that owners may reject the municipality interfering in tree removal decisions. This suggests that trees’ instrumental value is essential for residents in rural areas who see more provisioning benefits from trees.

Our research also shows the instrumental value of the trees and that the hidden relative values are important in shaping opinions surrounding who should decide to remove trees. These values can reinforce a relationship with the natural components of the environment and influence views regarding the management of natural properties, especially in rural areas.

Our research has some limitations. The first is lack of measurement of surrounding attachments to particular natural objects located directly in the territory of a person’s residence. Future research addressing tree removal views, need to look directly at attachments to natural objects such as trees or other elements of landscapes (Blanco et al., 2020). The second limitations is that we examine emotional attachment only to the place of residence. Further research should examine not only the strength of emotional attachment to one’s place of residence in forming an opinion about who should decide on tree removal but also attachment to vegetation or landscape on private property (Xu et al., 2019). Additionally, future research should focus on other dimensions of place attachment – e.g., place dependence, place identity (Williams and Vaske, 2003; Raymond et al., 2010; Anton and Lawrence, 2014), sense of place (Davenport and Anderson, 2005), social bonding (Kyle et al., 2005) in forming an opinion about who should decide on tree removal.

Results of our study, suggest that decision-makers regulating the removal of trees on privately owned land should consider the difference between rural and urban regions. This distinction may contribute to greater social acceptance of tree management across a regulatory regime. Decentralization of regulations and other environmental management considerations may avoid possible conflicts of interest in improving environmental management (Maczka et al., 2021). However, this study’s results refer to the cases of two municipalities, and there is a need for more extensive studies examining a larger number of cases.

The study results indicate that in tree management, it is crucial not only to perceive the value of trees but also to consider psychological variables related to the opinion of who should decide about tree removal, which is often missed in the preparation of programs on tree management. We recommend that in the future, stakeholders should cooperate with interdisciplinary teams, including psychologists, which will help to provide a holistic approach to solve the problem of tree management and the problem of conflicts between the administration and tree owners.

## Data Availability Statement

The datasets presented in this study can be found in online repositories. The names of the repository/repositories and accession number(s) can be found below: https://osf.io/ksbxw/.

## Ethics Statement

Ethical review and approval was not required for the study on human participants in accordance with the local legislation and institutional requirements. The patients/participants provided their written informed consent to participate in this study.

## Author Contributions

DP-M: conceptualization, literature review, methodology, validation, formal analysis, data curation, writing – original draft, visualization, and funding. PM: conceptualization, literature review, methodology, writing – review and editing, data collection, and funding. PJ: methodology, validation, formal analysis, data curation, and writing – original draft. All authors contributed to the article and approved the submitted version.

## Conflict of Interest

The authors declare that the research was conducted in the absence of any commercial or financial relationships that could be construed as a potential conflict of interest.
